# Predictors of Mortality in Pulmonary Hypertension-Associated Chronic Lung Disease

**DOI:** 10.3390/jcm13123472

**Published:** 2024-06-14

**Authors:** Jay Pescatore, Matthew Bittner, Gilbert D’Alonzo, Sheila Weaver, Shameek Gayen

**Affiliations:** Department of Thoracic Medicine and Surgery, Lewis Katz School of Medicine at Temple University Hospital, Philadelphia, PA 19140, USA; jay.pescatore@tuhs.temple.edu (J.P.); matthew.bittner@tuhs.temple.edu (M.B.); sheila.weaver@tuhs.temple.edu (S.W.)

**Keywords:** pulmonary hypertension, interstitial lung disease, chronic lung disease, pulmonary hemodynamics

## Abstract

**Background/Objectives**: Pulmonary hypertension (PH) often accompanies chronic lung diseases. Several chronic lung diseases with PH portends unfavorable outcomes. We investigated which variables in this cohort of patients with chronic lung disease and PH predicts mortality. **Methods**: This is a retrospective analysis of patients with chronic lung disease and PH at a single tertiary, academic center. The underlying lung disease included were COPD, IPF, other fibrotic ILD, non-fibrotic ILD, fibrotic sarcoidosis, and CPFE. All patients had right heart catheterization diagnostic of PH as well as pulmonary function testing data including 6 min walk testing. Univariable and multivariate Cox regression was performed to identify variables associated with mortality. **Results**: We identified 793 patients with chronic lung disease and PH. In total, 144 patients died prior to potential lung transplant. In multivariable Cox regression IPF, other fibrotic ILD, non-fibrotic ILD, and CPFE were significantly associated with an increased risk of mortality. Severe PH (PVR > 5 WU), FEV1 < 30% predicted, FVC < 40% predicted, 6 min walk distance < 150 m were also significantly associated with an increased risk of mortality. **Conclusions**: Carrying a diagnosis of IPF, CPFE, fibrotic ILD, or non-fibrotic ILD with PH has an increased risk of mortality as compared to COPD with PH. Hemodynamic, PVR > 5 WU, 6 min walk test less than 150 m, as well as spirometric data including FEV1 < 30% and FVC < 40% predicted were independently associated with an increased risk of death.

## 1. Introduction

Pulmonary hypertension (PH) often accompanies chronic lung diseases such as chronic obstructive pulmonary disease (COPD), idiopathic pulmonary fibrosis (IPF), pulmonary sarcoidosis, and other interstitial lung diseases (ILDs) [1]. The presence of PH in chronic lung disease portends unfavorable outcomes [2,3,4,5,6,7]. Notably, hemodynamic parameters may play a more integral role than lung function in long-term outcomes [8].

PH has been associated with an increased mortality in patients with COPD, IPF, combined pulmonary fibrosis, and emphysema (CPFE), in addition to other ILDs including connective tissue disease (CTD)-ILD and sarcoidosis [7,9,10,11,12,13,14]. Moreover, elevated pulmonary vascular resistance (PVR) is significantly associated with mortality including lung transplant waitlist mortality in patients with COPD, even when accounting for spirometry [4,15]. Beyond COPD, elevated PVR is also associated with increased mortality in patients with ILD and sarcoidosis [5]. The diagnosis of IPF, 6 min walk distance (6MWD), and reduced diffusing capacity of the lung for carbon monoxide (DLCO) may prognosticate patients with chronic lung disease and PH [3,13,16]. 

We hypothesize that mortality in patients with chronic lung disease and PH is predicted by factors related both to the underlying lung disease and severity of PH. Our primary objective is to determine significant and independent associations with mortality in patients with chronic lung disease and PH. 

## 2. Materials and Methods

### 2.1. Study Design 

We performed a retrospective analysis of patients with chronic lung disease (CLD) and PH at our institution referred for evaluation between 2011 and 2023; many but not all patients were referred for lung transplant evaluation. All patients were over 18 years of age. The underlying lung disease diagnoses were COPD, IPF, other fibrotic ILDs, non-fibrotic ILD, sarcoidosis, and CPFE. Underlying lung disease was diagnosed by pulmonary function testing and CT imaging. Clinical findings were interpreted and ultimately diagnosed by a pulmonologist. All patients with sarcoidosis or CTD-ILD had significant parenchymal involvement. All patients had a right-heart catheterization (RHC) diagnostic of PH. Patients were excluded if they did not have underlying parenchymal and/or spirometric lung disease with PH. The first RHC diagnosing PH was collected, with pulmonary function testing closest to the diagnostic RHC collected as well. New precapillary PH was defined as the mean pulmonary artery pressure (mPAP) > 20 mmHg with PVR > 2 Woods Units (WUs) and pulmonary arterial wedge pressure (PAWP) ≤ 15 mmHg as per the 2022 ERS/ESC guideline definitions [17]. Old pre-capillary PH was defined as PVR > 3 WU with the same mPAP and PAWP criteria [18]. New severe PH was defined as a PVR greater than 5 WU via the updated 2022 ESC guidelines [17]. Old severe PH was defined via the 2015 ESC/ERS guidelines with an mPAP greater than 35 mm Hg or greater than 25 mm Hg with a cardiac index of less than 2.5 L/min/m^2^ [18]. We evaluated pulmonary function testing data, specifically forced expiratory volume in one second (FEV1), forced vital capacity (FVC), and DLCO, which was performed at an accredited lab, and six-minute walk distance. FEV1 was stratified into standard parameters defined by GOLD COPD [19]. DLCO was stratified as severely reduced (<40% predicted) or not. 6MWD was stratified as <150 m, 150–300 m, or >300 m. Oxygen requirement during the six-minute walk test was stratified by the mean oxygen level in the cohort. We analyzed each patient’s 6 min walk distance and oxygen requirement with exertion. The outcomes collected were death prior to possible transplant, lung transplantation, alive without lung transplantation, and time from RHC to outcome. The authors used the Strengthening the Reporting of Observational Studies in Epidemiology (STROBE) checklist in the preparation of this manuscript. This study was performed in accordance with the ethical standards of the Helsinki Declaration of 1975 and Western Institutional Review Board (protocol # 29422). 

### 2.2. Statistical Analysis 

All continuous variables are presented as mean (standard deviation) unless otherwise stated. Categorical variables were compared using Pearson chi-squared test or the Fisher exact test where applicable. Continuous variables were compared between groups using the Mann–Whitney U test. Kaplan–Meier analysis was performed to evaluate the survival probability of the cohort. We performed univariable Cox regression to identify potential parameters (*p* < 0.1 in univariate Cox regression) associated with mortality prior to potential lung transplantation in our patient cohort. Following the identification of these potential parameters associated with our primary outcome of transplant-free survival, we performed a multivariable Cox regression analysis specifically with these parameters to identify the independent predictors of mortality in our patient cohort. Patients with missing data were included in the analysis and presented in the tables if significantly associated with the outcome. Statistical analysis was performed using IBM SPSS Statistics, version 25. 

## 3. Results

We identified 793 patients with lung disease and PH; demographic and baseline characteristics are provided in Table 1. Among these, 330 patients had underlying COPD, 130 patients had IPF, 121 patients had another fibrotic ILD, 37 patients had non fibrotic ILD, 70 patients had sarcoidosis with pulmonary fibrosis, and 105 patients had CPFE. 

The average age of the cohort was 63.3 years with a body mass index of 29.1. The male-to-female ratio was 1.03. Among the patients, 396 identified as Caucasians, 287 identified as black, and 110 identified as another race. Regarding comorbidities, 491 patients had chronic kidney disease, 260 had liver dysfunction, and 258 had diabetes.

Right-heart catheterization and pulmonary function testing upon initial evaluation were collected (Table 2). Among the patients, 273 patients met the prior 2015 ESC/ERS definition of severe pulmonary hypertension, while 248 patients met the updated 2022 ERS/ESC definition of severe PH [17,18]. Pulmonary functioning testing data were collected closest to the right-heart catheterization. Clinical characteristics stratified by lung disease are provided in the Supplementary Table S1. 

**Figure 1:**
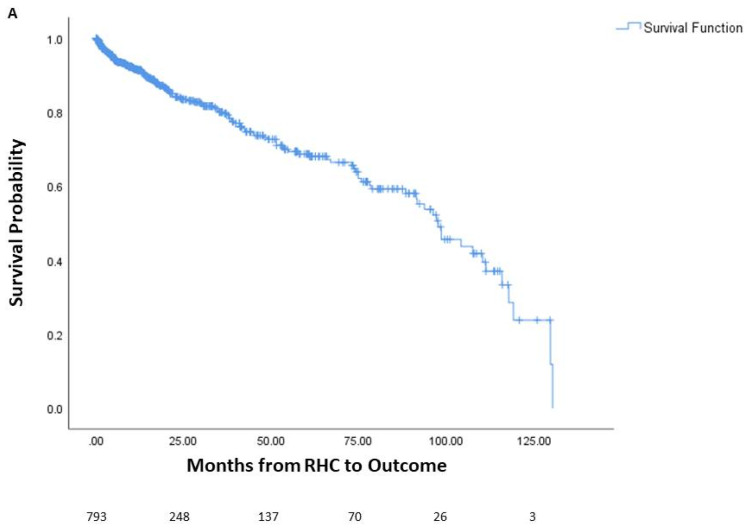

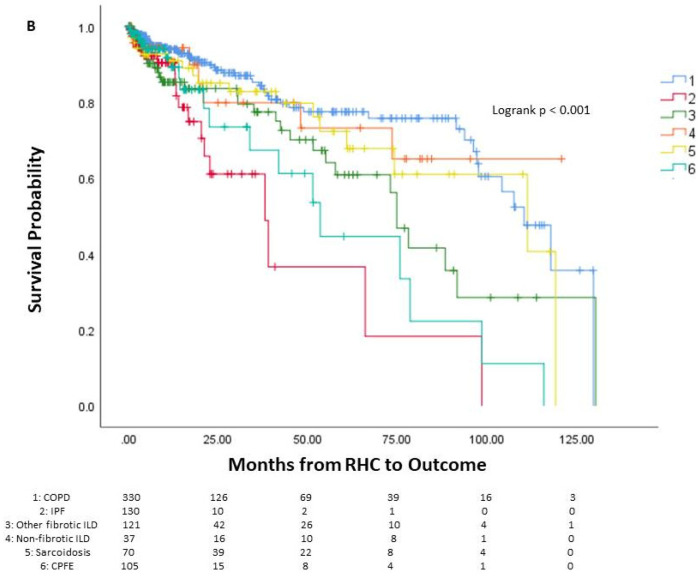
Survival of patients with chronic lung disease and pulmonary hypertension. Survival analysis of patients with chronic lung disease and pulmonary hypertension (**A**) and survival analysis stratified by underlying lung disease (**B**). The number of patients at risk is displayed at the bottom of the graph. In each graph, changes due to death before potential lung transplant are shown while those who received lung transplant are censored since they would no longer be at risk of death before transplantation. Lung transplantation is not reflected in the actual Kaplan–Meier survival curves. RHC: right-heart catheterization.

Among the patients, 144 died prior to potential lung transplant, 436 underwent lung transplantation, and 213 were alive without lung transplantation. The average time from right heart catheterization to outcome was 24.5 months, or 2.04 years. One-year mortality was 6.9% (55 patients), three-year mortality was 11.5% (91 patients), and five-year mortality was 14.4% (114 patients). The survival curve of the cohort is seen in Figure 1, along with survival curves stratified by underlying lung disease. A significant difference in survival probability was seen between the various lung diseases (logrank *p* < 0.001), with COPD patients having the best survival probability and IPF patients having the worst survival probability.

Univariate Cox regression was performed to identify the variables associated with mortality before transplant, with variables showing a trend towards significant association (*p* < 0.1) and subsequently multivariable Cox regression to determine the independent and significant predictors of mortality (Table 3). Underlying lung disease, old criteria for pre-capillary PH, old criteria for severe PH, new criteria for severe PH, FEV1, FVC, 6MWD, and oxygen requirement were identified as potential parameters (*p* < 0.1) in univariate Cox regression and subsequently utilized in the multivariable Cox regression. IPF (HR 5.09, 95% CI 2.56–10.13, *p* < 0.001), other fibrotic lung disease (HR 2.65, 95% CI 1.45–4.84, *p* = 0.002), non-fibrotic lung disease (HR 2.48, 95% CI 1.10–4.55, *p* = 0.03), and CPFE (HR 3.14, 95% CI 1.53–6.43, *p* = 0.002) were significantly and independently associated with an increased risk of mortality. The new criteria for severe PH (PVR > 5 WU; HR 2.10, 95% CI 1.21–3.63, *p* = 0.01), FEV1 < 30% predicted (HR 2.23, 95% CI 1.03–3.55, *p* = 0.02), FVC < 40% predicted (HR 1.51, 95% CI 1.28–1.93, *p* = 0.03), and 6 min walk distance less than 150 m (HR 2.18, 95% CI 1.27–3.75, *p* = 0.01) were also significantly and independently associated with an increased risk of mortality. 

## 4. Discussion

Among patients with chronic lung disease and PH, we elucidated the following factors that independently and significantly predicted increased mortality: underlying lung disease, severe PH as defined by PVR > 5 WU, reduced 6MWD, reduced FEV1, and reduced FVC. Specifically, the diagnoses of IPF, other ILDs, and CPFE were associated with increased mortality among this patient cohort. Our findings suggest that mortality among patients with chronic lung disease and PH is driven by pulmonary hemodynamics, underlying lung disease, and lung function. 

The latest 2022 ESC/ERS guidelines for the diagnosis and management of PH define severe PH in chronic lung disease as PVR > 5 WU, shifting from the previous mPAP-driven definition [17]. This change in definition results from studies demonstrating that a PVR threshold of 5 WU is a better predictor of mortality in patients with COPD-PH and ILD-PH [4,6]. This finding was also found in patients with sarcoidosis-associated PH [5]. Severe PH as defined by the 2022 ESC/ERS guidelines was a strong independent predictor of mortality in our cohort of patients, even when accounting for underlying lung disease and lung function. Notably, the older, mPAP-driven definition of severe PH was not significantly associated with increased mortality among this cohort. This crucial finding further substantiates PVR greater than 5 WU, as a better prognosticator of PH than mPAP. It also proves PVR > 5 WU to be a more substantial marker of mortality across several chronic lung diseases, not only in sarcoid-associated PH, COPD-PH, and ILD-PH as previously found. 

We also elucidated factors beyond pulmonary hemodynamics impacting mortality in our cohort, even when accounting for the severity of the PH. Notably, the underlying diagnoses of IPF, CPFE, and other ILDs, both fibrotic and non-fibrotic, significantly increase the risk of mortality as compared to COPD. Among the patients with chronic lung disease with parenchymal involvement and PH, those with COPD have demonstrated better survival [20]. As such, we used COPD as our reference lung disease, and the risk of mortality for each lung disease was as compared to COPD. Other studies have demonstrated the importance of underlying lung disease as well. Alhamad et al. found that among patients with parenchymal lung disease and concomitant PH, the presence of IPF independently correlated with increased mortality; notably, this study did not include patients with COPD or CPFE [3]. Likewise, in our cohort we found that IPF was significantly associated with mortality, specifically having the highest risk of death among all lung diseases. While IPF patients tend to have a more severe and progressive clinical course compared to other lung diseases, some studies suggest that patients with CPFE may fare even worse. Patients with IPF and concomitant emphysema have been found to have worse outcomes than those with IPF alone, an outcome at least in part associated with the development of severe PH [21]. It is important to note that CPFE carries an increased risk of PH prevalence and severity as compared to IPF and COPD, further suggesting that poor outcomes in CPFE-PH compared to other lung diseases is in part driven by PH severity [14]. However, we notably found that underlying lung disease was predictive of increased mortality independently of pulmonary hemodynamics, suggesting that, even in those with PH, underlying lung disease is important for prognostication. Additionally, our finding of increased risk of mortality in non-fibrotic ILD in the spectrum of underlying lung disease is novel as well. 

We found that lung function determined via spirometry holds prognostic value among patients with chronic lung disease and PH, namely FEV1 < 30% predicted and FVC < 40% predicted. FEV1 represents an essential variable in the risk stratification of patients with COPD and is used in the Global Initiative for Chronic Obstructive Lung Disease severity staging for COPD [19]. FEV1 also serves to predict mortality, acting as one of the variables that independently predicts mortality in COPD as comprised within the BODE index (body mass index, airflow obstruction, dyspnea, exercise capacity) [22,23]. FVC has demonstrated prognostic significance among various restrictive lung diseases. Progressive decline in FVC is predictive of mortality in IPF, while a low baseline FVC is predictive of early mortality in systemic sclerosis [24,25]. As such, FVC is a key variable in studies of fibrotic lung disease trials, such as the PROGRESS and INPULSIS trials [26,27]. The PROGRESS Trial investigated the progressing fibrosing phenotype of ILD and found that a decline in FVC of more than 10% was strongly associated with an increased risk of death. The INPULSIS trial investigated the antifibrotic Nintedanib for IPF, using the annual rate of decline in FVC as their primary end point. Notably, the prognostic significance of spirometry has not been widely demonstrated among patients with chronic lung disease and PH but is not altogether surprising. It is crucial that these variables were validated in our cohort of patients with PH encompassing both obstructive and restrictive lung diseases, as again it demonstrates that more than pulmonary hemodynamics drive these patients’ symptoms and mortality. This key pathophysiologic dynamic between the pulmonary vascular and the parenchyma is seen in both chronic progressive lung disease as well as in acute lung diseases. For example, Rossi et al. investigated the development of PH and right ventricular dysfunction among COVID-19 survivors, highlighting how acute interstitial pneumonias can trigger pulmonary vascular changes [28]. Such pulmonary vascular changes are likely more pronounced and prolonged in chronic lung diseases.

The 6-min walk distance is a strong predictor of outcomes for various lung diseases including IPF, COPD, sarcoidosis, and for group 1 pulmonary arterial hypertension [29,30,31,32,33]. It is also used as a common endpoint for clinical trials, pertinent to this patient population, such as the INCREASE trial, as well as in risk scores for COPD, including the BODE index [23,34]. In addition to prognostication value, 6MWD also provides fruitful information regarding functional status, oxygenation with exercise, and hemodynamic response to exercise—all factors reflective of the severity of underlying parenchymal and pulmonary vascular disease. Thus, while not unexpected, our cohort also validated the importance of a reduced 6-min walk distance in predicting mortality. 

Surprisingly, we did not find that a severely reduced DLCO, defined as <40% predicted, to be associated with mortality, despite the literature that supports this parameter in both chronic lung disease and pulmonary hypertension [16,35,36]. There are several potential reasons as to why we did not find a similar significance. Many patients in the cohort had a severely reduced DLCO of less than 40% predicted, which could mask identifying significance. Additionally, many of the studies that found a significant association between DLCO and mortality used a lower cutoff of 35% predicted (or lower), which may facilitate finding a true difference in mortality outcomes. We used less than 40% predicted as that is the clinical definition for severely reduced DLCO. 

To our knowledge, this study is the first to investigate such a wide array of chronic lung diseases with PH and evaluate the independent variables impacting mortality, which we believe is a strength of our study. The majority of other studies examining patients with chronic lung disease and PH focused on a subset of lung diseases rather than the wide array captured in our study. We were able to capture both hemodynamic and lung disease parameters that predicted the mortality of chronic lung disease patients with PH. The large sample size of our cohort as a whole and in each underlying lung disease is another strength of our study. A few other groups have developed risk stratification scores for mortality in ILD-PH [37,38]. However, it is unclear which of the variables within this scoring system are the predominant drivers of mortality. Moreover, none of these studies have endeavored to encompass such a wide range of CLD as we have in our current study. 

The limitations of this study include its retrospective nature as well as the fact that it encompasses only one academic healthcare center. Our data collection was limited by information available within the electronic medical record. The timing of RHC and pulmonary function testing was not protocolized. Our study design was cross-sectional, thus limiting our ability to infer causality. Moreover, our patient population is somewhat unique, even amongst those with chronic lung disease and PH. Many of our patients were referred for lung transplant evaluation, suggesting a higher degree of severity of their pulmonary parenchymal and vascular disease. However, at the same time, patients undergoing lung transplant evaluation tend to have fewer comorbid conditions than the general population. Given these considerations, our findings may not be fully applicable to a more general population of chronic lung disease and PH patients. However, our findings are still significant and for the most part are in line with previous findings, while encompassing a more diverse and potentially “sicker” patient population. Additionally, while some patients were prescribed pulmonary vasodilator therapy, we did not include this in our analysis as we were focused on patient characteristics that predict mortality, rather than interventions. We acknowledge this is a limitation to our retrospective study. Further studies, ideally prospective and multicenter in nature, are needed to validate our findings to help reduce bias, improve the accuracy of the data, and generalizability.

In conclusion, IPF, CPFE, fibrotic lung disease, and non-fibrotic ILD have an increased risk of mortality compared to COPD. Severe PH defined as PVR > 5 WU, 6MWD less than 150 m, as well as spirometric data FEV1 < 30% and FVC < 40% predicted were associated with an increased risk of death. These findings can assist in prognosticating patients with chronic lung disease and pulmonary hypertension and aid in referral to a tertiary care center including for lung transplantation. This leads us to the future direction of our study, which is to create a risk assessment tool that encompasses several chronic lung disease diagnoses, degree of pulmonary vascular disease impairment, and pulmonary parenchymal impairment. 

## Figures and Tables

**Table 1:** Demographics and population comorbidities, (*n*= 793).

**Mean age** (SD)	63.3 (9.9)
**Mean body mass index** (SD)	29.1 (7.1)
**Gender**	
Male, *n* (%)	403 (50.8%)
Female, *n* (%)	390 (49.2%)
**Race/ethnicity**	
Non-Hispanic black, *n* (%)	287 (36.2%)
Non-Hispanic white, *n* (%)	396 (49.9%)
Other, *n* (%)	110 (13.9%)
**Population Comorbidities**	
**Underlying lung disease**	
COPD, *n* (%)	330 (41.6%)
IPF, *n* (%)	130 (16.4%)
Other fibrotic ILD, *n* (%)	121 (15.3%)
Non-fibrotic ILD, *n* (%)	37 (4.7%)
Fibrotic sarcoidosis, *n* (%)	70 (8.8%)
CPFE, *n* (%)	105 (13.2%)
**Comorbidities**	
CKD, *n* (%)	491 (61.9%)
Liver dysfunction, *n* (%)	260 (32.7%)
Diabetes, *n* (%)	258 (32.5%)

COPD, chronic obstructive respiratory disease; IPF, idiopathic pulmonary fibrosis; ILD, interstitial lung disease; CPFE, combined pulmonary fibrosis and emphysema; CKD, chronic kidney disease.

**Table 2:** Clinical characteristics.

Right-heart catheterization	
Right atrial pressure, mean (SD)	7.2 mmHg (4.7)
Pulmonary artery systolic pressure, mean (SD)	48.8 mmHg (16.7)
Mean pulmonary artery pressure, mean (SD)	31.2 mmHg (10.1)
Pulmonary capillary wedge pressure, mean (SD)	12.5 mmHg (10.7)
Cardiac output, mean (SD)	5.0 L/Min (2.7)
Cardiac index, mean (SD)	2.6 L/Min/m^2^ (0.7)
Old pre-capillary PH, *n* (%)	429 patients (54%)
New pre-capillary PH, *n* (%)	575 patients (73%)
Old severe PH, *n* (%)	273 patients (34%)
New severe PH, *n* (%)	248 patients (31%)
Pulmonary function tests	
% Predicted FEV1, mean (SD)	47% (24.1)
% Predicted FEV1 > 80%, *n* (%)	79 patients (10.0%)
% Predicted FEV1 50–80%, *n* (%)	277 patients (28.6%)
% Predicted FEV1 30–50%, *n* (%)	211 patients (14.0%)
% Predicted FEV1 < 30%, *n* (%)	189 patients (23.8%)
% Predicted FVC, mean (SD)	60.1% (23.3)
% Predicted FVC > 80%, *n* (%)	152 patients (19.2%)
% Predicted FVC 40–80%, *n* (%)	521 patients (65.7%)
% Predicted FVC < 40%, *n* (%)	82 patients (10.3%)
% Predicted DLCO, mean (SD)	28.1% (15.2)
DLCO < 40%, *n* (%)	407 patients (51.3%)
6-min walk distance, mean (SD)	239.3 m (96.2)
>300 m, *n* (%)	93 patients (11.7%)
150–300 m, *n* (%)	479 patients (60.4%)
>150 m, *n* (%)	93 patients (11.7%)
Oxygen requirement on exertion, mean (SD)	5.5 L/min (4.9)
>5 L/min, *n* (%)	404 patients (50.9%)
BNP, mean (SD)	453.7 pg/dL (868.2)

BNP, B-type natriuretic peptide; FEV1, forced expiratory volume in one second; FVC, forced vital capacity; DLCO, diffuse capacity of carbon monoxide.

**Table 3:** Univariate and multivariable Cox regression.

Variable	Univariable Cox Regression	Multivariable Cox Regression
Underlying lung disease ^a,b^		
IPF	HR 3.25, 95% CI 1.91–5.54, *p* < 0.001	**HR 5.09, 95% CI 2.56–10.13, *p* < 0.001**
Other fibrotic ILD	HR 1.91, 95% CI 1.21–3.02, *p* = 0.005	**HR 2.65, 95% CI 1.45–4.84, *p* = 0.002**
Non-fibrotic ILD	HR 1.11, 95% CI 0.50–2.46, *p* = 0.80	**HR 2.48, 95% CI 1.10–4.55, *p* = 0.03**
Sarcoidosis	HR 1.34, 95% CI 0.77–2.33, *p* = 0.30	HR 1.85, 95% CI 0.96–3.55, *p* = 0.07
CPFE	HR 2.48, 95% CI 1.45–4.24, *p* = 0.001	**HR 3.14, 95% CI 1.53–6.43, *p* = 0.002**
Pre-capillary PH (old definition) ^a^	HR 1.44, 95% CI 1.03–2.02, *p* = 0.03	HR 0.78, 95% CI 0.49–1.25, *p* = 0.30
Pre-capillary PH (new definition)	HR 1.24, 95% CI 0.86–1.78, *p* = 0.25	
Cardiac index	HR 0.89, 95% CI 0.68–1.15, *p* = 0.36	
Severe PH (2015 definition) ^a^	HR 1.80, 95% CI 1.29–2.51, *p* = 0.001	HR 1.09, 95% CI 0.67–1.78, *p* = 0.73
Severe PH (2022 definition) ^a,b^	HR 2.30, 95% CI 1.65–3.21, *p* < 0.001	**HR 2.10, 95% CI 1.21–3.63, *p* = 0.01**
FEV1 (>80% pred is reference) ^a,b^		
50–80% predicted	HR 0.56, 95% CI 0.32–0.98, *p* = 0.04	HR 1.23, 95% CI 0.58–2.63, *p* = 0.59
30–50% predicted	HR 0.76, 95% CI 0.43–1.34, *p* = 0.34	HR 1.85, 95% CI 0.79–4.33, *p* = 0.16
<30% predicted	HR 0.54, 95% CI 0.29–1.00, *p* = 0.05	**HR 2.23, 95% CI 1.03–3.55, *p* = 0.02**
FVC (>80% pred is reference) ^a,b^		
40–80% predicted	HR 0.70, 95% CI 0.46–1.05, *p* = 0.09	HR 0.60, 95% CI 0.24–4.33, *p* = 0.29
<40% predicted	HR 1.15, 95% CI 0.59–2.09, *p* = 0.73	**HR 1.51, 95% CI 1.28–1.93, *p* = 0.03**
DLCO < 40% predicted	HR 1.59, 95% CI 0.97–2.62, *p* = 0.07	HR 1.10, 95% CI 0.61–1.97, *p* = 0.76
6MWD (>300 m is reference) ^a,b^		
150–300 m	HR 1.19, 95% CI 0.81–1.75, *p* = 0.37	HR 0.94, 95% CI 0.60–1.47, *p* = 0.78
<150 m	HR 1.89, 95% CI 1.12–3.17, *p* = 0.02	**HR 2.18, 95% CI 1.27–3.75, *p* = 0.01**
Oxygen requirement > 5 L ^a^	HR 0.70, 95% CI 0.48–1.02, *p* = 0.06	HR 0.79, 95% CI 0.52–1.20, *p* = 0.26
BNP	HR 1.00, 95% CI 0.98–1.01, *p* = 0.10	

^a^ Variable demonstrated trend towards significant association with an outcome of mortality prior to potential transplantation in univariable Cox regression (*p* < 0.1) and included in multivariable Cox regression. ^b^ Variable demonstrated an independent and significant association with the outcome of mortality prior to possible transplantation in multivariable Cox regression analysis. BNP, B-type natriuretic peptide; IPF, idiopathic pulmonary fibrosis; ILD, interstitial lung disease; CPFE, combined pulmonary fibrosis and emphysema; FEV1, forced expiratory volume in one second; FVC, force vital capacity; DLCO, diffuse capacity of carbon monoxide; 6MWD, 6-min walk distance.

## Data Availability

The data that support the findings of this study are available from the corresponding author upon reasonable request.

## Supplementary Materials

The following supporting information can be downloaded at: https://www.mdpi.com/article/10.3390/jcm13123472/s1, Table S1: Clinical characteristics by underlying lung disease.
