# Supplementary material for: Predictors of Mortality in Pulmonary Hypertension-Associated Chronic Lung Disease
Source: J Clin Med. 2024 Jun 14;13(12):3472. doi: 10.3390/jcm13123472 (PMC11205208; doi:10.3390/jcm13123472)
Supplement: Supplementary file 1 [file jcm-13-03472-s001.zip › jcm-3059861-supplementary.pdf]

| **Supplemental Table S1**: Clinical Characteristics by Underlying Lung Disease | | | | | | |
|---|---|---|---|---|---|---|
| **Variable[a]** | **COPD** | **IPF** | **Other Fibrotic ILD** | **Non-Fibrotic ILD** | **Sarcoidosis** | **CPFE** |
| **RA Pressure** | 8.6 mmHg (4.4) | 4.4 mmHg (4.1) | 6.3 mmHg (4.1) | 8.3 mmHg (5.7) | 7.9 mmHg (4.7) | 5.9 mmHg (4.9) |
| **Systolic PA Pressure** | 46.2 mmHg (16) | 47.2 mmHg (15) | 50.9 mmHg (17.4) | 54.6 mmHg (20.7) | 55 mmHg (16.9) | 50.2 mmHg (16.5) |
| **Mean PA Pressure** | 30.5 mmHg (9.7) | 28.7 mmHg (8.7) | 31.8 mmHg (10) | 34 mmhg (12.3) | 35.5 mmHg (10.2) | 31.6 mmHg (10.9) |
| **PCWP** | 13.6 mmHg (6) | 10.2 mmHg (5.6) | 13.4 mmHg (23.3) | 13 mmHg (8) | 13.5 mmHg (6.4) | 10.3 mmHg (5.1) |
| **Cardiac Output** | 4.8 L/min (1.4) | 4.9 L/min (1.3) | 5.5 L/min (3.9) | 5.1 L/min (1.5) | 5.5 L/min (6.4) | 4.9 L/Min (1.4) |
| **Cardiac Index** | 2.6 L/min/m2 (0.7) | 2.5 L/min/m2 (0.6) | 2.7 L/min/m2) (0.8) | 2.6 L/min/m2 (0.7)) | 2.4 L/min/m2 (0.7) | 2.5 L/min/m2 (0.7) |
| **PVR** | 4.9 WU (11.9) | 4.7 WU (3.8) | 4.8 WU (3.8) | 5.4 WU (4.9) | 5.8 WU (3.6) | 5 WU (3.2) |
| **FEV1 % Predicted** | 34% (20.9) | 57.9% (20) | 53.2% (18.8) | 50.3% (28.9) | 50.8% (22.4) | 63% (22.1) |
| **FVC % Predicted** | 62% (24.8) | 53.9% (20.2) | 52.4% (18.3) | 53.2% (27.7) | 59% (21.3) | 70% (21.2) |
| **DLCO % Predicted** | 29.6% (14.7) | 22.6% (15.7) | 26.8% (13.5) | 40.9% (19.4) | 33.9% (14.4) | 22.3 % (12.1) |
| **Oxygen Requirement on Exertion** | 4.5 L/min (3.8) | 7.4 L/min (6.3) | 6.3 L/min (4.7) | 4.3 L/min (6.3) | 4 L/min (3.5) | 7.1 L/min (5.2) |
| **6 MWD** | 224.7 m (87.7) | 255.1 m (100) | 244.7 m (119.3) | 274.8 m (86) | 255.2 m (85) | 238.2 m (91.6) |

[a]Variables calculated in mean
COPD, Chronic Obstructive Respiratory Disease; IPF, Idiopathic Pulmonary Fibrosis; ILD, Interstitial Lung Disease; Combined Pulmonary Fibrosis and Emphysema; Chronic Kidney Disease; RA, Right Atrium; PA,Pulmonary Artery; PCWP, Pulmonary Capillary Wedge Pressure; PVR, Pulmonary Vascular Resistance; WU, Woods Units; FEV1, Forced Expiratory Volume in One Second; FVC, Forced Vital Capacity; DLCO, Diffuse Capacity of Carbon Monoxide; MWD, Minute Walk Distance
